# Hypoglycemic and hypolipidemic activity of combined milk thistle and fenugreek seeds in alloxan-induced diabetic albino rats

**DOI:** 10.14202/vetworld.2020.1732-1736

**Published:** 2020-08-29

**Authors:** Mohamed Jamal Saadh

**Affiliations:** Department of Pharmacy, Faculty of Pharmacy, Middle East University, Amman, Jordan

**Keywords:** fenugreek seeds, hypoglycemic activity, hypolipidemic activity, milk thistle seeds

## Abstract

**Background and Aim:**

Despite the availability of antidiabetic drugs, they are not free from associated adverse side effects. This study aimed to evaluate the hypoglycemic and hypolipidemic effects of oral administration of seeds from two medicinal plants: (1) Milk thistle and (2) fenugreek.

**Materials and Methods:**

Plant seeds were washed in distilled water and ground with a coffee grinder. Alloxan was used to induce diabetes in 20 male albino rats. Diabetic rats were randomly divided into two groups: (1) Group 1 (n=10), diabetic rats fed with 0.5 g/kg milk thistle and 2 g/kg fenugreek seeds per day and (2) Group 2 (n=10), diabetic rats fed standard rodent food for 4 weeks.

**Results:**

Oral administration of milk thistle and fenugreek seeds for 2 weeks resulted in significant improvement in body weight, blood glucose, glycosylated hemoglobin (HbA1c), cholesterol, and triglyceride levels in alloxan-induced diabetic rats. After 4 weeks, this ameliorative effect was significantly elevated with respect to blood glucose (155.00±9.70 mg/dL vs. 427.50±5.70 mg/dL; p<0.001), HbA1c (5.5±0.19% vs. 13.65±1.77%; p<0.001), cholesterol (281.50±10.95 mg/dL vs. 334.30±6.80 mg/dL; p<0.001), triglyceride (239.60±6.87 mg/dL vs. 284.20±9.95 mg/dL; p<0.01), and body weight (265.30±8.10 g vs. 207.40±11.4 g; p<0.01) as compared with non-treated diabetic rats.

**Conclusion:**

Milk thistle and fenugreek seeds possess hypoglycemic and hypolipidemic properties and could be used as natural compounds that are suitable as parent compounds for the development of new antidiabetic drugs.

## Introduction

Diabetes mellitus (DM) is a chronic complex metabolic disorder that occurs in response to complete or insufficient cessation of insulin secretion or synthesis and/or insulin peripheral resistance causing disturbances in carbohydrate, proteins, and fat metabolism [1]. An estimated 425 million adults worldwide have DM, and this number is predicted to rise to 629 million by 2045. This increase in the prevalence of DM will cause large social and economic burden, especially in low- to middle-income countries, where about 75% of people with DM live [2].

Although different types of antidiabetic drugs are available and most are effective in providing long-term glycemic control [1], they are not free from some associated adverse side effects such as flatulence, cramps, diarrhea, nausea, and gastrointestinal irritation [3]. In addition, prolonged use of these drugs results in a response deficiency [4]. For example, after 6 years of sulfonylurea treatment, the effectiveness of the drug is insufficient in 44% of patients [5]. Thus, there is an urgent need to explore options that include traditional medicinal plants with no side effects for DM management.

*Silybum marianum*, or milk thistle, belongs to the family *Carduus marianum* and has been known for more than 2000 years to be an herbal remedy used for a variety of disorders [6]. The components of this plant scavenge free radicals to protect the body against oxidative peroxidation. In addition to its antioxidant, anti-inflammatory, and anticancer properties, it is regarded as a potent agent against diabetes-induced hyperglycemia and insulin resistance [7]. In addition, fenugreek, which belongs to the *Fabaceae* family, is reported to have neuroprotective, antioxidant, antilithogenic potential, antihyperlipidemic, and a stimulating/regenerating effect on β-cells and antidiabetic effects [8,9]. The hypoglycemic properties of fenugreek seeds have been demonstrated in experimentally induced diabetic rats, diabetic patients, and healthy volunteers [8,10]. Taken together, medicinal plants are useful dietary supplements to existing therapies as well as provide oral antidiabetic bioactive compounds for new pharmaceutical development [8]. Although various kinds of medicinal plants have been reported to have hypoglycemic and hypolipidemic effects, these plants have failed to achieve greater effectiveness.

Therefore, the aim of this study was to investigate the effect of oral administration of combined milk thistle with fenugreek seeds on body weight, blood glucose, glycosylated hemoglobin (HbA1c), cholesterol, and triglyceride levels of alloxan-induced diabetic rats.

## Materials and Methods

### Ethical approval

All animal experiments were performed in accordance with the guidelines of the National Council for Animal Experimentation Control and the Ethical Committee approval was obtained from ethical committee of Middle East University, Jordan.

### Study period and location

The study was conducted from May 2019 to December 2019 at Middle East University, Amman, Jordan.

### Plant material

Fenugreek seeds were purchased from a local supermarket of Syrian origin, and milk thistle was purchased from Frontier Herbs. Seeds were washed in distilled water and ground with a coffee grinder to an average particle diameter of 0.3 mm.

### Animals and induction of experimental diabetes

Twenty male albino rats weighing between 250 and 300 g were obtained from the Faculty of Pharmacy, Middle East University, Amman, Jordan. Before initiating the experiments, rats were fed standard rodent food for 1 week for acclimation to the laboratory conditions. Diabetes induction in these rats was carried out 4 weeks before the start of the experiment. Immediately before use, alloxan monohydrate (Sigma-Aldrich Chemical) was dissolved in sterile normal saline. The dose of alloxan for 160 mg/kg body weight was injected intraperitoneally to all male adult albino rats after 6 h. For the next 24 h, the rats were maintained on 5% glucose solution bottles in their cages to prevent hypoglycemia [11]. Fasting blood glucose values >7 mmol/L (126 mg/dL) were considered diabetic [12].

### Experimental design

Before the diabetic rats were feed milk thistle and fenugreek seeds, their body weight was recorded and blood samples were collected from the tail vein to estimate blood glucose, HbA1c, cholesterol, and triglyceride levels. The 20 diabetic rats were randomly divided into two groups (10 rats/group) as follows: Group 1 (n=10), diabetic rats fed daily with 0.5 g milk thistle and 2 g fenugreek seeds per 1 kg of body weight per day (~0.5 g fenugreek/rat with 0.125 g milk thistle/rat/day) for 4 weeks by mixing the ground fenugreek and milk thistle with digestive biscuits (sugar free), which were prepared as dough (1.5 g) and Group 2 (n=10), diabetic rats fed standard rodent food without milk thistle and fenugreek seeds for 4 weeks. The body weight of the rats was recorded at the 2^nd^ week and at the end of the experiment (4^th^ week), and blood samples were collected to measure glucose, HbA1c, cholesterol, and triglyceride levels.

### Blood collection

Blood samples were collected from the tail vein, during which a large volume of blood (up to 2 mL/withdrawal) was drawn. Briefly, local anesthetic cream was applied to the surface of the tail for 30 min, and then, the tail was dipped into warm water (40°C). A 23G syringe with a needle was inserted into the vein, and blood was collected in the EDTA Vacutainer tubes [13].

### HbA1c test

HbA1c was determined using whole EDTA blood. In the test samples, HbA1c was absorbed onto the surface of the latex particles, which reacted with anti-HbA1c (antigen-antibody reaction) to provide agglutination. The amount of agglutination was measured as the absorbance. The HbA1c value was obtained from a calibration curve. The procedure is described in the insert (Spectrum, Egypt).

### Biochemical analysis

Plasma was separated by centrifugation at 3000 rpm for 10 min and then subjected to biochemical analysis. The plasma sample was used for the quantitative determination of glucose using the enzymatic colorimetric method (glucose oxidase-peroxidase), of cholesterol using the enzymatic colorimetric method (PAP), and of triglycerides using the enzymatic colorimetric method (glycerol-3-phosphate oxidase) level in blood using commercial kits (Arcomex, Jordan). The procedure followed the instructions described in the kits [14].

### Statistical analysis

Data were presented as mean±SD of three parallel measurements. Statistical significance was assessed by *t-*test (using two-tailed distribution) using SPSS software version 20.0 (SPSS Inc., Chicago, IL). p<0.05 was set as statistically significant.

## Results

**Figure-1:**
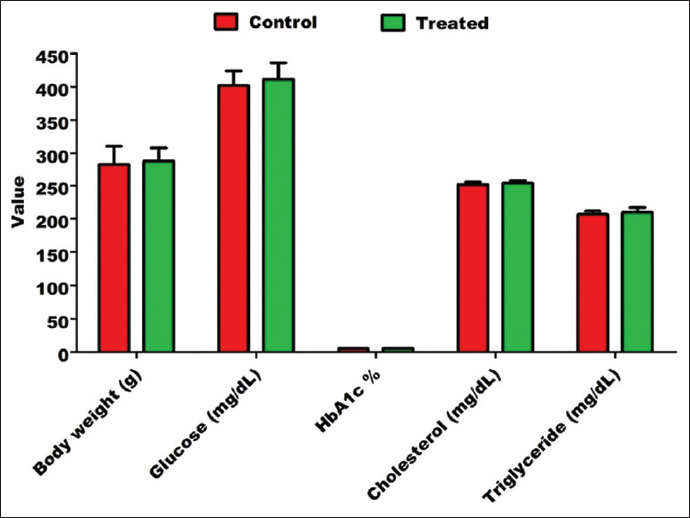
Body weight, blood glucose, glycosylated hemoglobin, serum cholesterol, and serum triglyceride in alloxan-induced diabetic rats. In pre-treatment.

**Figure-2:**
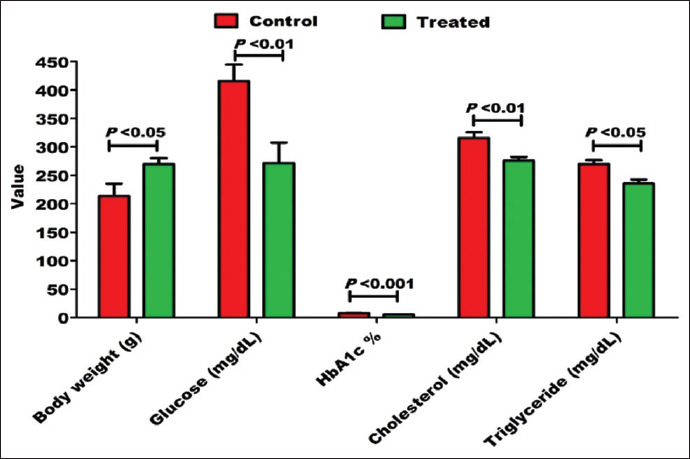
After 2 weeks of oral administration of 0.5 g/kg milk thistle and 2 g/kg fenugreek seeds per day, there was a significant improvement in the body weight, blood glucose, glycosylated hemoglobin, serum cholesterol, and serum triglyceride in alloxan-induced diabetic rats.

Diabetes induction with alloxan was associated with body weight loss and elevated levels of glucose, HbA1c, cholesterol, and triglyceride levels. In contrast to pre-treatment findings (Figure-1), oral administration of 0.5 g/kg milk thistle and 2 g/kg fenugreek seeds per day (~0.5 g fenugreek/rat with 0.125 g milk thistle/rat per day) for 2 weeks resulted in a significant reduction in blood glucose (271.80±35.60 vs. 415.80±29.10 mg/dL; p<0.01), HbA1c (5.21±0.35% vs. 7.61±0.4%; p<0.001), cholesterol (275.90±6.50 vs. 316.20±9.80 mg/dL; p<0.01), and triglyceride (235.70±6.86 vs. 270.10±6.99 mg/dL; p<0.05) levels in alloxan-induced diabetic rats and led to a significant improvement in the body weight of diabetic rats (270.20±9.90 vs. 213.70±21.70 g; p<0.05; Figure-2; Table-1).

**Table-1:** Changes in body weight, blood glucose, HbA1c, serum cholesterol, and serum triglyceride in alloxan-induced diabetic rats after oral administration of 0.5 g/kg milk thistle and 2 g/kg fenugreek seeds per day.

Loss of weight		Diabetes tests				Lipid tests			
		
Body weight (g)		Glucose (mg/dL)		HbA1c (%)		Cholesterol (mg/dL)		Triglyceride (mg/dL)	
		
C	T	C	T	C	T	C	T	C	T
Changes after 2 weeks (%)									
−24.50	−6.30	+3.50	−33.90	+55.30	+5.89	+25.90	+8.70	+30.70	+12.20
Changes after 4 weeks (%)									
−26.80	−8.00	+6.40	−62.30	+178.60	+11.70	+32.40	+10.90	+37.60	+14.10

C=Control, T=Treated, +=Increase, −=Decrease. Significantly different from control by *P<0.05, **P<0.01, ***P<0.001, HbA1c=Glycosylated hemoglobin

**Figure-3:**
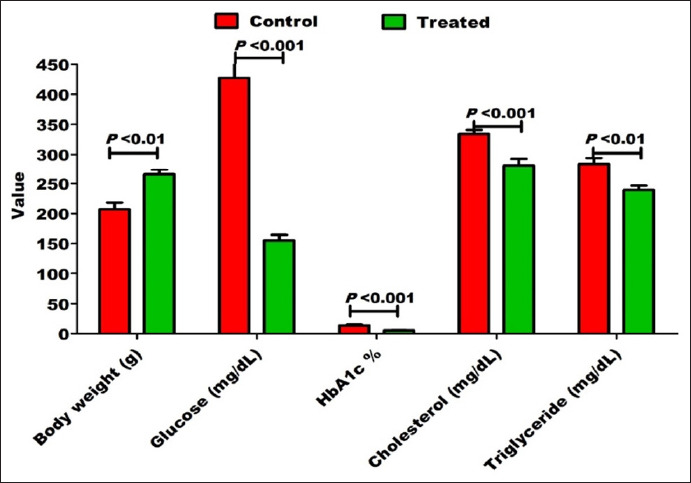
After 4 weeks of oral administration of 0.5 g/kg milk thistle and 2 g/kg fenugreek seeds per day, there was a significant improvement in the body weight, blood glucose, glycosylated hemoglobin, serum cholesterol, and serum triglyceride in alloxan-induced diabetic rats.

After 4 weeks of oral administration of seeds, this ameliorative effect was significantly elevated for blood glucose (155.00±9.70 vs. 427.50±5.70 mg/dL; p<0.001), HbA1c (5.50±0.19% vs. 13.65±1.77%; p<0.001), cholesterol (281.50±10.95 vs. 334.30±6.80 mg/dL; p<0.001), triglyceride (239.60±6.87 vs. 284.20±9.95 mg/dL; p<0.01), and body weight (265.30±8.10 vs. 207.40±11.40 g; p<0.01) of diabetic rats (Figure-3; Table-1). Table-2 presents the decrease or increase in the percentage of body weight, glucose, HbA1c, cholesterol, and triglycerides in the treated diabetic rats as compared with the diabetic control group.

**Table-2:** Decrease or increase the percentage of body weight, glucose, HbA1c, cholesterol, and triglyceride in treated diabetic rats comparing with non-treated diabetic control group.

Loss of weight	Diabetes tests		Lipid tests	
		
Body weight (g)	Glucose (mg/dL)	HbA1c (%)	Cholesterol (mg/dL)	Triglyceride (mg/dL)
After 2 weeks (%)				
+26.40	−34.63	−31.53	−12.74	−12.73
After 4 weeks (%)				
+27.90	−63.70	−59.70	−15.79	−15.69

+=Increase, −=Decrease, HbA1c=Glycosylated hemoglobin

## Discussion

DM is a growing health problem in most countries. It is a major and chronic endocrine disorder caused by acquired and/or inherited deficiency in insulin production by the pancreas or by secreted insulin ineffectiveness. In addition, DM is associated with many complications, such as neuropathy, kidney disease, retinopathy, and heart disease [15]. Although several drugs are used to reduce hyperglycemia, such as α-glucosidase inhibitors, metformin, and sulfonylureas, diabetes and its linked complications still constitute important medical problems [16]. Many natural medicinal plants and herbs have strong antidiabetic properties and are safe, relatively non-toxic, and even free from serious side effects [9].

Results of this study revealed that in alloxan-induced diabetic rats, oral administration of 0.5 g/kg milk thistle and 2 g/kg fenugreek seeds per day for 2 weeks caused a significant reduction in blood glucose, HbA1c, cholesterol, and triglyceride levels and a significant improvement the body weight. This ameliorative effect was significantly elevated after 4 weeks of oral administration of seeds. These results further suggest that milk thistle and fenugreek seeds could be used as a potential treatment for diabetes.

The results of many studies using diabetic experimental models have demonstrated that milk thistle plant seeds or extracts have an overall beneficial effect on weight, glucose level, and lipid profile [17-19]. It was suggested that by triggering the gut–brain–liver axis, milk thistle has functional potential as an antidiabetic food ingredient. In this study, findings revealed a significant inhibition of blood glucose in diabetic rats administrated milk thistle for 4 weeks. In addition, the activation of neurons in the nucleus of the solitary tract and expression of glucagon-like peptide-1 receptor in the duodenum increased, whereas hepatic glucose production decreased after milk thistle administration [19].

On the other hand, peroxisome proliferator-activated receptor γ (PPAR-γ), the molecular target of thiazolidinediones, is used clinically as an insulin sensitizer to lower blood glucose levels in diabetic patients. A substance from milk thistle has been shown to possess PPAR-γ agonist properties. Studies indicated that partial PPAR-γ agonism induces promising activity patterns by retaining the positive effects attributed to the full agonists, with reduced side effects [6].

Moreover, in hypercholesterolemic rats, treatment with milk thistle had a significant ameliorative effect on lipid profile, and it decreased both serum and hepatic total cholesterol, triglycerides, very low-density lipoprotein cholesterol, and low-density lipoprotein cholesterol and increased high-density lipoprotein cholesterol [17]. Milk thistle has also demonstrated beneficial effects on several diabetic complications, including non-alcoholic steatohepatitis, diabetic nephropathy, and diabetic neuropathy mainly by means of its antioxidant properties [6].

Preliminary human [20,21] and animal [22-24] trials have suggested the possible hypoglycemic effect and antihyperlipidemic properties of oral fenugreek seeds. Fenugreek seeds have also previously been shown to have hypocholesterolemic and hypoglycemic effects on diabetic patients and experimental diabetic animals [25].

Like insulin, fenugreek also induces phosphorylation of the insulin tyrosine kinase receptor in adipocytes and liver cells [26]. In addition, the circulating antioxidant activity of fenugreek has been reported through the significant decrease in lipid peroxide level, which exerts beneficial effects on the increased oxidative stress in diabetic patients [27,28]. Phytochemical screening demonstrated that fenugreek contains trigocoumarin, trigonelline, and trimecoumarine alkaloids, which have antihyperglycemic effects [28]. Fenugreek seed fibers also decrease the glucose absorption rate and may delay gastric emptying, thereby preventing a rise in blood glucose levels after a meal [29]. In diabetic patients, fenugreek guar gum prevents the rapid uptake of glucose in the small intestine, aids in blood glucoses retention, and may also be effective in the treatment of hypercholesterolemia [30]. Moreover, seed fibers contain an amino acid, 4-hydroxyisoleucine, that stimulates insulin secretion, because the cells are more sensitive to insulin, and increases the number of insulin receptor sites to burn cellular glucose [31]. Fenugreek seed extracts have also been reported to exhibit antidiabetic potential by protecting β-cells and restoring the function of pancreatic tissue, elevating the serum insulin level, possibly through stimulation of insulin release from existing β-cells of islets or by β-cell regeneration, and stimulating glycogen synthetase activity [9].

## Conclusion

Our study demonstrated that the oral administration of dried milk thistle and fenugreek seeds is associated with hypoglycemic and hypolipidemic effects and they can be used as natural compounds suitable for the development of new antidiabetic drugs.

## Author’s Contributions

MS designed the study, drafting the manuscript, performed all the experimental procedures, and conducted data analysis and interpretation. The author read and approved the final version of the manuscript.

## Competing Interests

The author declares that he has no competing interests.

## Publisher’s Note

Veterinary World remains neutral with regard to jurisdictional claims in published institutional affiliation.

## References

[1] Adams D.M, Yakubu M.T. (2020). Aqueous extract of *Digitaria exilis* grains ameliorate diabetes in streptozotocin-induced diabetic male Wistar rats. J. Ethnopharmacol.

[2] Gomes M.B, Rathmann W, Charbonnel B, Khunti K, Kosiborod M, Nicolucci A, Pocock S.J, Shestakova M.V, Shimomura I, Tang F, Watada H, Chen H, Cid-Ruzafa J, Fenici P, Hammar N, Surmont F, Ji L. (2019). Treatment of Type 2 diabetes mellitus worldwide: Baseline patient characteristics in the global DISCOVER study. Diabetes Res. Clin. Pract.

[3] Nayak B.S, Marshall J.R, Isitor G, Adogwa A. (2011). Hypoglycemic and hepatoprotective activity of fermented fruit juice of *Morinda citrifolia* (Noni) in diabetic rats. Evid. Based Complement. Alternat. Med.

[4] Mukherjee P.K, Maiti K, Mukherjee K, Houghton P.J. (2006). Leads from Indian medicinal plants with hypoglycemic potentials. J. Ethnopharmacol.

[5] Verma S, Singh S.P. (2008). Current and future status of herbal medicines. Vet. World.

[6] Kazazis C.E, Evangelopoulos A.A, Kollas A, Vallianou N.G. (2014). The therapeutic potential of milk thistle in diabetes. Rev. Diabet. Stud.

[7] Arvanag F.M, Bayrami A, Habibi-Yangjeh A, Pouran S.R. (2019). A comprehensive study on antidiabetic and antibacterial activities of ZnO nanoparticles biosynthesized using *Silybum marianum* L seed extract. Mater. Sci. Eng. C.

[8] Geberemeskel G.A, Debebe Y.G, Nguse N.A. (2019). Antidiabetic effect of fenugreek seed powder solution (*Trigonella foenum-graecum* L.) on hyperlipidemia in diabetic patients. J. Diabetes Res.

[9] Mooventhan A, Nivethitha L. (2017). A narrative review on evidence-based antidiabetic effect of fenugreek (*Trigonella foenum-graecum*). Int. J. Nutr.

[10] Srinivasan K (2006). Fenugreek (*Trigonella foenum-graecum*): A review of health beneficial physiological effects. Food Rev. Int.

[11] Gupta M.P, Solis N.G, Avella M.E, Sanchez C. (1984). Hypoglycemic activity of *Neurolaena lobata* (L.) R. BR. J. Ethnopharmacol.

[12] Castro K.M.R, de Paiva Carvalho R.L, Junior G.M.R, Tavares B.A, Simionato L.H, Bortoluci C.H.F, Soto C.A.T, Ferraresi C. (2020). Can photobiomodulation therapy (PBMT) control blood glucose levels and alter muscle glycogen synthesis?. J. Photochem. Photobiol. B Biol.

[13] Parasuraman S, Raveendran R, Kesavan R. (2010). Blood sample collection in small laboratory animals. J. Pharmacol. Pharmacother.

[14] Tietz N.W (1976). Fundamentals of Clinical Chemistry.

[15] Devi P.R.S, Reddy A.G, Rao G.S, Kumar C.S.V, Boobalan G. (2015). Pharmacokinetic interaction of curcumin and glibenclamide in diabetic rats. Vet. World.

[16] Oluwafemi O, Oguntibeju O.O. (2019). Medicinal plants and their effects on diabetic wound healing. Vet. World.

[17] Gobalakrishnan S, Asirvatham S.S, Janarthanam V. (2016). Effect of silybin on lipid profile in hypercholesterolaemic rats. J. Clin. Diagn. Res.

[18] Sajedianfard J, Behroozi Z, Nazifi S. (2014). The effects of a hydroalcoholic extract of silymarin on serum lipids profiles in streptozotocin induced diabetic rats. Comp. Clin. Pathol.

[19] Xu F, Yang J, Negishi H, Sun Y, Li D, Zhang X, Hayashi T, Gao M, Ikeda K, Ikejima T. (2018). Silibinin decreases hepatic glucose production through the activation of gut-brain-liver axis in diabetic rats. Food Funct.

[20] Haritha C, Reddy A.G, Reddy Y.R, Anilkumar B. (2015). Pharmacodynamic interaction of fenugreek, insulin and glimepiride on sero-biochemical parameters in diabetic Sprague-Dawley rats. Vet. World.

[21] Sharma R.D, Raghuram T.C, Rao N.S. (1990). Effect of fenugreek seeds on blood glucose and serum lipids in Type I diabetes. Eur. J. Clin. Nutr.

[22] Gaddam A, Galla C, Thummisetti S, Marikanty R.K, Palanisamy U.D, Rao P.V. (2015). Role of fenugreek in the prevention of Type 2 diabetes mellitus in prediabetes. J. Diabetes Metab. Disord.

[23] Mishra N (2013). Haematological and hypoglycemic potential *Anethum graveolens* seeds extract in normal and diabetic Swiss albino mice. Vet. World.

[24] Riyad M.A, Abdul-Salam S.A, Mohammad S.S. (1988). Effect of fenugreek and lupine seeds on the development of experimental diabetes in rats. Planta Med.

[25] Xue W.L, Li X.S, Zhang J, Liu Y.H, Wang Z.L, Zhang R.J. (2007). Effect of *Trigonella foenum-graecum* (fenugreek) extract on blood glucose, blood lipid and hemorheological properties in streptozotocin-induced diabetic rats. Asia Pac. J. Clin. Nutr.

[26] Vijayakumar M.V, Singh S, Chhipa R.R, Bhat M.K. (2005). The hypoglycemic activity of fenugreek seed extract is mediated through the stimulation of an insulin signaling pathway. Br. J. Pharmacol.

[27] Devasena T, Menon V.P. (2002). Enhancement of circulatory antioxidants by fenugreek during 1,2-dimethylhydrazine-induced rat colon carcinogenesis. J. Biochem. Mol. Biol. Biophys.

[28] Mowla A, Alauddin M, Rahman M.A, Ahmed K. (2009). Antihyperglycemic effect of *Trigonella foenum-graecum* (fenugreek) seed extract in alloxan-induced diabetic rats and its use in diabetes mellitus:A brief qualitative phytochemical and acute toxicity test on the extract. Afr. J. Tradit. Complement. Altern. Med.

[29] Gupta A, Gupta R, Lal B. (2001). Effect of *Trigonella foenum-graecum* (fenugreek) seeds on glycaemic control and insulin resistance in Type 2 diabetes mellitus: A double blind placebo controlled study. J. Assoc. Physicians India.

[30] Bruce-Keller A.J, Richard A.J, Fernandez-Kim S.O, Ribnicky D.M, Salbaum J.M, Newman S, Carmouche R, Stephens J.M. (2020). Fenugreek counters the effects of high fat diet on gut microbiota in mice:Links to metabolic benefit. Sci. Rep.

[31] Broca C, Manteghetti M, Gross R, Baissac Y, Jacob M, Petit P, Sauvaire Y, Ribes G. (2000). 4-Hydroxyisoleucine:Effects of synthetic and natural analogues on insulin secretion. Eur. J. Pharmacol.

